# Immunobiology of Solid Cancers: Cellular and Molecular Pathways as Potential Diagnostic and Therapeutic Targets 

**DOI:** 10.1155/2018/6532019

**Published:** 2018-03-01

**Authors:** Ilary Ruscito, Elena Ioana Braicu, Maria Luisa Gasparri, Ilaria Grazia Zizzari

**Affiliations:** ^1^UP Cell Therapy and Tumor Immunology, Department of Experimental Medicine, Sapienza University of Rome, Viale Regina Elena 324, 00161 Rome, Italy; ^2^Tumorbank Ovarian Cancer Network (TOC), Department of Gynecology, Charité-Universitätsmedizin Berlin, Corporate Member of Freie Universität Berlin, Humboldt-Universität zu Berlin, and Berlin Institute of Health, Augustenburger Platz 1, 13353 Berlin, Germany; ^3^Department of Obstetrics and Gynecology, University Hospital of Bern and University of Bern, Effingerstrasse 102, 3010 Bern, Switzerland; ^4^Department of Gynecology, Obstetrics and Urology, Sapienza University of Rome, Viale del Policlinico 155, 00161 Rome, Italy; ^5^Department of Medical and Surgical Sciences and Translational Medicine, Sapienza University of Rome, Via di Grottarossa 1035, 00189 Rome, Italy

In the last four decades, tumor immunology has shed light on identity and functions of cells and molecules involved in tumor rejection through the involvement of the immune system [1]. Several groups of immune cells have been demonstrated to be able to contrast tumor occurrence and tumor progression by killing immunogenic tumor cells, a phenomenon recognized under the definition of “immunosurveillance” [2]. Unfortunately, cancer may evade immunosurveillance and progress through the modifications of its own antigens, which can reduce tumor immunogenicity and/or increase its immunosuppressive action [3]. After years of investigations, harnessing the immune system to attack cancer has recently led scientists to gather enough clinical data to show what a powerful sword immunotherapy can be [4]. Data on unexpected clinical recoveries and long progression-free intervals are increasing regarding patients addressed to immunotherapy treatments [5, 6]. Despite its extraordinary success, only a portion of cancer types and cancer patients benefit from immunotherapy treatments. Understanding the reason why this happens is the big challenge of our time and, in order to answer this question, basic science is crucial: to elucidate how tumor cells and immune cells interact with each other in cancer patients and clarify the mechanisms through which tumor mutational pattern affects the response to therapies is the way to pursue for improving efficacy of current treatments and promoting new anticancer strategies. This special issue was conceived with the aim of collecting new findings in the field of cancer immunology and describing novel biological and molecular evidence on the relationship between cancer and immune system as well as cancer and immunotherapy.

In response to the aim of the special issue, four original research papers and one review article are presented below.

The study reported by B. Cerbelli et al. from Sapienza University of Rome, Italy, showed that immunohistochemical PD-L1 expression in ≥25% of triple negative breast cancer (TNBC) chemo-naïve cells, derived from core biopsies, is an independent predictor for pathological complete response (pCR) after neoadjuvant chemotherapy, thus discussing potentials and limits of PD-L1 future applications as a predictive biomarker for neoadjuvant treatment response in this subset of patients affected by such a clinically aggressive disease.

M. Moschetta et al., at Sarah Cannon Research Institute of London, UK, presented a study assessing the impact of “neutrophil-to-lymphocyte ratio” (NLR) in predicting PFS among 55 advanced patients enrolled into PD-1/PD-L1 inhibitors phase 1 clinical trials. Results showed a significant longer PFS in patients with a reduction of NLR after two treatment cycles compared to the median baseline NLR, thus advancing the hypothesis that NLR may be a helpful predicting tool in cancer patients treated with anti-PD-1/PD-L1 agents.

An international collaboration between USA (University of Colorado and Yale School of Medicine) and Japan (Kindai University and Chugai Pharmaceutical), coordinated by K. Suda et al., obtained evidence concerning molecular mechanism behind the low expression of PD-1/PD-L1 in NSCLC, associated with reduced efficacy of checkpoint inhibitors (CI) treatments. The study highlighted that EGFR-mutated lung cancer cell lines do not show high PD-L1 expression and, furthermore, after acquisition of resistance to EGFR-TKIs, EGFR phosphorylation affects PD-L1 expression, thus identifying a molecular event able to influence the expression of biomarkers, which regulate patients' access to CI agents.

Apart from its immunomodulatory function, F. Zheng et al., from Huazhong University of Science and Technology, China, identify PD-L1 molecule as a potential biomarker of melanoma cancer stem-like cells, since blocking PD-L1 in melanoma cell lines expressing PD-L1 and ALDH1 impaired tumorsphere formation and induced the apoptosis of tumorsphere cells. These findings raise the need to elucidate the relationship between tumor response to checkpoint inhibitors and clonal evolution of cancer stem cells in the future.

Finally, the review paper by M. Wei et al., Southeast University of China, discusses the role of gastric cancer patients' T cells immunity and disease prognosis, providing a critical synthesis of recent evidence on this still controversial topic.

In conclusion, we find this special issue to be a good opportunity for improving knowledge in the field of cancer immunobiology and immunotherapy, which is a pivotal step to respond adequately to the questions of our time in the battle against cancer.

*Ilary Ruscito* *Elena Ioana Braicu* *Maria Luisa Gasparri* *Ilaria Grazia Zizzari*
